# Tannin-Based Copolymer Resins: Synthesis and Characterization by Solid State ^13^C NMR and FT-IR Spectroscopy

**DOI:** 10.3390/polym9060223

**Published:** 2017-06-13

**Authors:** Gianluca Tondi

**Affiliations:** Forest Product Technology & Timber Construction Department, Salzburg University of Applied Sciences, Marktstraße 136a, 5431 Kuchl, Austria; gianluca.tondi@fh-salzburg.ac.at; Tel.: +43-50-2211-2250

**Keywords:** natural macromolecules, flavonoids, black wattle, curing, green chemistry, nuclear magnetic resonance, infrared

## Abstract

In recent years, the interest for bio-sources is rising exponentially and tannins extracts are one of the most interesting, easily-available, phenolic building blocks. The condensed tannins or proanthocyanidins are already known for their polymerization chemistry, which is the basis for several natural-based materials (e.g., adhesives, foams). In the present work we aim to observe the behavior of the extract of Acacia Mimosa (Acacia mearnsii) when reacted with several possible co-monomers at different relative amount, pH and temperature conditions. The more insoluble copolymers obtained with formaldehyde, hexamine, glyoxal, maleic anhydride, furfural and furfuryl alcohol were analyzed through solid state ^13^C NMR (Nuclear magnetic resonance) and FT-IR (Fourier Transform-Infrared) spectroscopy. The ^13^C NMR afforded the opportunity to detect: (i) aromatic substitutions and consequent poly-condensations for the majority of the hardeners studied; (ii) acylation for the maleic anhydride and also some; (iii) Diels–Alder arrangements for the furanic co-monomers; the FT-IR spectroscopy suggested that the formaldehyde and hexamine copolymers present a higher cross-linking degree.

## 1. Introduction

Plastic is a common name for many different materials and the great majority of them are synthetic. That is principally because is not easy to find natural alternative resources that can guarantee the same performance as synthetic polymers. In some cases, the use of natural polymers is possible because nature has already synthesized them in a way that can be exploited by humans. For instance, wool is mainly natural proteins synthesized directly by sheep, and cotton is a polymer of cellulose produced by a plant [1,2]. In both cases we are just the final users of these materials, applying physical treatment to obtain the requested properties. In some cases, we modify the natural polymers chemically to have the opportunity to reuse them in different formations, such as in the case of viscose [3]. It is much more complicated to produce polymers from bio-resources which originally have a lower molecular mass. In this case, polymerization reactions are requested to produce macromolecules, and the condensed tannins represent one of the few natural phenolic fractions which is suited to this purpose, without the need of heavy activation processes [4,5,6,7,8].

**Figure 1:**
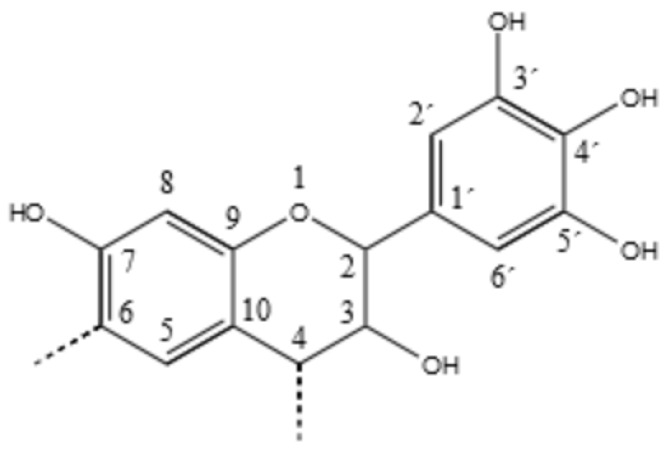
Prorobinetinidin structure: Resorcinolic A-ring and pyrogallic B-ring.

Tannin is the common name for wood extracts containing a high amount of polyphenols, that can fix the proteins of the animal hide to produce leather [9,10]. These polyphenols are produced by superior plants to protect the ligno-cellulosic material against biological and radiative degradation, due to their chemistry and their antioxidant capacity [11,12,13]. Chemically they can be classified into two major families: condensed, and hydrolysable tannins [14]. Condensed tannins are made of proanthocyanidin, of which the four main building blocks are: Profisetidin, procyanidin, prorobinetinidin and prodelphinidin [15]. These molecules and their oligomers are characterized by having similar reactivity to that of phenol in the polymerization with formaldehyde. In Figure 1, the chemical structure of the prorobinetinidin oligomer, which is the predominant flavonoid in the mimosa tannin, is reported [16,17].

According to the different chemical reactions possible, several molecules were selected for the hardening of the tannin extract: (i) The “known” hardeners: formaldehyde and hexamine [18,19,20]; (ii) The dialdehydes, such as glyoxal, glutaraldehyde and *t*-phthaldialdehyde [21]; (iii) the nitrogen-containing compounds such as dicyandiamide, caprolactam and chitosan [22]; (iv) the di-poly functional acid/anhydrides, such as citric and phthalic acid, and maleic anhydride [23] and finally (v) the furanic compounds [24].

The main goal of the present research was to produce a set of tannin-based polymers in different conditions of pH (between 2.0 and 9.0) and temperature (from 20 to 103 °C) and determine their water resistances. Solid state ^13^C NMR and FT-IR of the insoluble fractions were performed to obtain information about the chemical structure of these macromolecules.

## 2. Materials and Methods

### 2.1. Materials

Mimosa (Acacia Mearnsii) tannin extract was provided by the company Silva team (S. Michele Mondoví, Italy). Hexamethylentetramine (Hexamine), glyoxal (sol. 40%) and maleic anhydride from Merck (Darmstadt, Germany); furfural from Lenzing (Lenzing, Austria); furfuryl alcohol from Transfuran chemicals (Geel, Belgium); formaldehyde (sol. > 35%), glutaraldehyde, citric acid and phthalic acid from Roth (Karlsruhe, Germany); caprolactam, dicyandiamide from Aldrich (St. Louis, MO, USA) were used as possible comonomers while sulphuric acid from Roth and sodium hydroxide from Merck were used to modify the pH.

### 2.2. Methods

#### 2.2.1. Formulation Preparation and Hardening

A wide set of tannin formulations were prepared in plastic test tubes of 50 mL volume; different amounts of hardeners were added to 20 g of a 33.3% solution of tannin in water at specific pH (2, 4.5, 7 and 9) levels. Table 1 shows the hardeners and the amounts in relation to the tannin, and finally, the pH. The acid pH was obtained by adding H_2_SO_4_ (33%); the pH of 4.5 is the value of the tannin solution, and the pH of 7 and 9 were obtained by adding a 33% water solution of NaOH. Once the formulations were completed, the plastic containers were sealed with screw caps, vigorously mixed and exposed to increasing temperature. The samples were stored successively at 20, 50, 70, 90 and 103 °C, keeping the samples 24 h for every temperature. The hardening temperature was registered for every sample when the test tube presented a solid block.

The solids were removed from the test tube after that the last heating phase (103 °C, 24 h) was concluded. They were firstly broken and then preliminarily grinded, before drying them at 60 °C for 24 h, in order to allow water and volatile compounds to evaporate. The materials obtained were further finely grinded, with pestle and mortar, until the powders presented homogeneous granulometry (<300 µm) and stored again at 60 °C for a further 24 h.

#### 2.2.2. Leachability Tests

One gram of dry powder was then immersed in 100 mL of deionized water at 20 °C, and left under stirring for 1 h. The suspension was then filtered with a previously dried and weighed filter paper of 125 µm. The filter containing the insoluble was dried at 103 °C for 24 h. The dry weight of the powder was registered and the percentage of insoluble was reported. These tests were repeated three times per powder.

The insoluble leached powders of the polymer with 25% of hardener and with the pH which guaranteed the lower solubility (Formaldehyde pH = 4.5; Hexamine pH = 9; Glyoxal pH = 4.5; Maleic anhydride pH = 4.5; Furfural pH = 9; Furfuryl alcohol pH = 2) were then analyzed by solid state ^13^C NMR and FT-MIR.

#### 2.2.3. ^13^C NMR Analysis

Seven powders were measured with the solid state ^13^C NMR spectrometer, Bruker Avance III HD 400 Spectrometer (Bruker BioSpin, Rheinstetten, Germany), at the Chemistry department of the University of Natural Resources and Life Sciences of Vienna. The samples were measured with a 4 mm dual-broadband CP-MAS probe with a frequency of 100.66 MHz and acquired by TOSS (total sideband suppression) at ambient temperature with a spinning rate of 5 kHz. The chemical shifts were referenced externally against the carbonyl signal of glycine with δ = 176.03 ppm [25].

The calculations of the theoretical chemical shifts were done with the software NMR Predict developed by the University of Lausanne (Luc Patiny) and the University of del Valle (Julien Wist) [26,27,28].

#### 2.2.4. FT-IR Investigation

The same seven powders were analyzed with the Frontier ATR-FT-MIR from Perkin-Elmer (Waltham, MA, USA). Every powder was scanned three times, with 16 scans from 4000 to 600 cm^−1^ and the fingerprint spectral region between 1800 and 600 cm^−1^ was selected. Then the spectra were normalized, baseline corrected and averaged with the Unscrambler software by CAMO (Oslo, Norway).

#### 2.2.5. Principal Component Analysis (PCA)

The Unscrambler software was also applied to the study of the FT-IR spectra of the tannin-based hardened copolymers. It was performed applying the validation method NIPALS (NonLinear Iterative Partial Least Squares) on a full model size in the spectral region between 1800 and 600 cm^−1^.

## 3. Results and Discussion

### 3.1. Copolymer Preparation

A wide range of tests were performed in order to determine which combination carried the most interesting materials and the principal findings are reported in Table 2.

The table shows that 4% of formaldehyde s./s. already allows for the the polymerization of tannin. The favorite pHs are pH 2 and 9, when the polymer occurs already at room temperature. However, all the formulations cure when the temperature rises at pH 4.5 and 7 too. This observation is in line with previous studies [15].

Hexamine is also a very efficient hardener for the Mimosa tannin, but in this case, the pH plays a major role. The tannin–hexamine formulation remain homogeneous only when the pH is alkaline or neutral. At pH 9, the polymer is homogeneous and occurs all over the solution, while at pH 7, there is a significant amount of unreacted material. The polymerization temperature varies between 50 °C for a high pH and high amount at 90 °C. Conversely, when the pH is acidic, the tannin complex with hexamine immediately produces a clear lumpy solid at room temperature, which has already been noticed elsewhere [29].

The group of the aldehydes shows that only glyoxal produces a solid which requires at least 90 °C to cure, and the polymers produced are generally elastic. Glutaraldehyde produces a weak gel and *p*-phthaldialdehyde produces a two-phase solution at pH 9. Both hardeners do not show significant evidence of polymerization and therefore, they will not be considered any further.

The nitrogen containing compounds, caprolactam, chitosan and dicyandiamide, do not allow for the curing of tannin. Only chitosan at low pH produces some lumps similar to one produced by hexamine at these pH levels. None of these hardeners will be considered further.

In the group of the di-functional acids, the only one offering an interesting curing process is the maleic anhydride. Indeed, the tannin–maleic anhydride solutions produce elastic materials similar to the glyoxal with various percentages of hardener. Conversely, citric and phthalic acid tannin solutions remain liquid.

Finally, the group of furanics show several polymerization possibilities. Furfural produces solids from pH 4.5 to 9.0, with a significant amount of hardener. The higher the pH, the lower the curing temperature observed. The furfuryl alcohol only produces polymers at pH 2 and high temperatures. The polymers of these two hardeners will be both analyzed.

In summary, the most promising hardeners were formaldehyde, hexamine, glyoxal, maleic anhydride, furfural and furfuryl alcohol, and their tannin polymers, which then underwent water leaching.

### 3.2. Leaching Resistance

**Figure 2:**
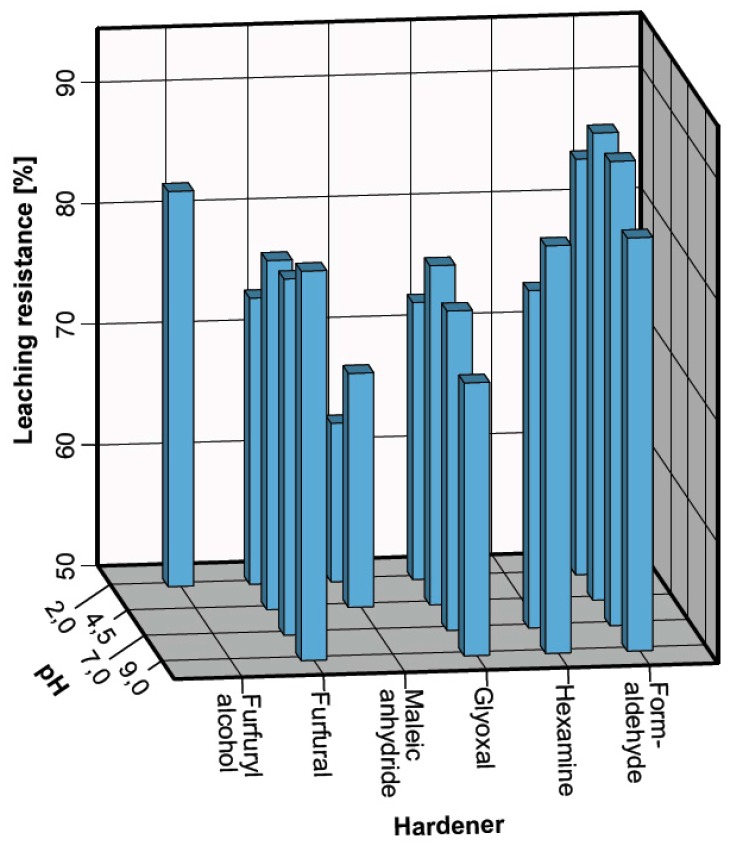
Leaching resistance of the tannin polymers with different hardeners and pH.

The solids identified after curing as potential tannin–polymers were tested for their leaching resistance, and the results registered are summarized in Figure 2.

It can be seen that every hardener presents its maximal water resistance at different pHs: Formaldehyde produces its more insoluble polymer at pH 4.5 and 7.0 (88.63% and 88.38%, respectively). This result was unexpected because the tannin–formaldehyde polymers at pH 2.0 and 9.0 occurs at lower temperature. This could be explained by a more compacted arrangement for the molecules, which become solid at higher temperature. Hexamine polymers have a higher leaching resistance at pH 9.0 (83.71%). Glyoxal–tannin polymers cure almost simultaneously between 90 and 103 °C, but also in this case the polymer produced at pH 4.5 produces more insoluble results (78.05%). Tannin–Maleic anhydride polymers occur only when the pH is very low. The formulation with the original pH of 4.5 produces results that are more resistant against leaching (69.34%); it has to be noted that 25% of maleic anhydride (*w/w* tan) contributes to significantly decreasing the pH value (1.8) of the formulation. Tannin–furfural polymers are more easily produced at alkaline pH (9.0) and in this case, these polymers produce results that are also more insoluble (82.20%). Furfuryl alcohol reacts with tannin only in acidic environments, and the leaching resistance observed for the solid produced at pH 2.0 was 82.19%.

The polymers which presented higher leaching resistances were Formaldehyde (4.5), Hexamine (9.0), Glyoxal (4.5), Maleic anhydride (4.5), Furfural (9.0), and Furfuryl alcohol (2.0). These were analysed by ^13^C NMR and FT-IR to determine the chemistry which explains these results, by considering the leached polymers with excess hardener (25%), in order to maximize the spectroscopic evidences.

### 3.3. ^13^C NMR Investigation

The chemical information of a solid state ^13^C NMR spectra of the tannin–polymers can be divided in 4 spectral regions. The region between 190 and 140 ppm of the C_Arom_ directly bonded with oxygen (C_Arom_–O); the region between 140 and 90 ppm of the C_Arom_ did not directly connect with oxygen (C_Arom_–C and C_Arom_–H); the region between 90 and 50 of C_aliph_ directly bonded with oxygen (C_Aliph_–O) and the region 50–10 ppm of the C_aliph_ did not directly bond with an oxygen (C_Aliph_–C and C_Aliph_–H).

#### 3.3.1. Tannin–Formaldehyde (pH = 4.5)

**Figure 3:**
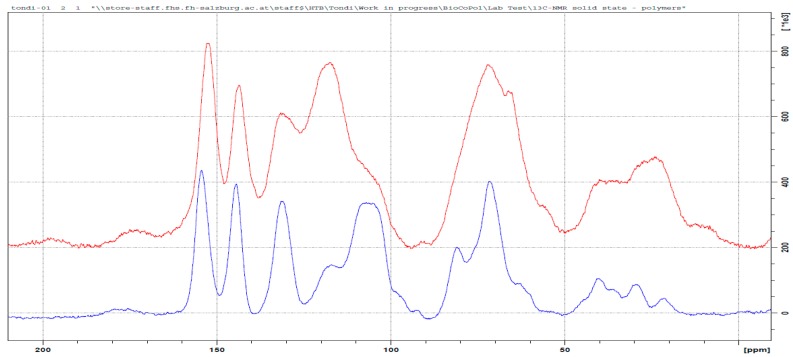
Solid state ^13^C NMR of the original tannin extract (blue) and the leached tannin–formaldehyde polymer (red).

In Figure 3, the solid state ^13^C NMR spectrum of the tannin–formaldehyde polymer in comparison with that of the industrial tannin extract, is reported.

The polymerization chemistry of phenols, which is certainly the most known, is that with formaldehyde [14,30]. Condensed tannins have similar reactivity to that of phenol and therefore, it is expected that its polymerization follows similar patterns involving a methylol activation of the resorcinol ring (ring A), which evolves into methylene bridges (φ–CH_2_–φ) [31] and/or to intermediate (di)methylene-ether bridges (φ–CH_2_–O–CH_2_–φ) [32,33]. The regions of the methylene-ether bridge (~70 ppm) and that of the methylene bridge (~30 ppm) result both highly increased with a major impact for the methylene-ether bridges. Chains of methylene-ethers groups, such as φ–CH_2_–O–CH_2_–O–CH_2_–φ and free *p*-formaldehyde, can be excluded due to the absence of signals between 90 and 100 ppm, while the presence of unreacted methylol groups (–CH_2_–OH) can be observed in the small amount at 55 ppm. These methylol branches can oxide to aldehyde and to acid, and the two small signals at 195 and 175 ppm confirm this statement. The ^13^C NMR spectrum of the tannin–formaldehyde polymer presents major modifications in the region between 140 and 90 ppm (C_Arom_ region). The more significant band increase occurs at around 120 ppm, and the major decrease occurs at 105 ppm. These two absorptions are strongly interconnected because they are related to the –OH free aromatic positions of both the resorcinolic ring (C5, C6, C8 and C10) and the pyrogallic (C1’, C2’ and C6’) ring of the prorobinetinidin [34]. When these positions are substituted, they absorb at higher frequencies (lower magnetic fields). The positions available for being activated are C5, C8 (or C6) and C2’and C6’. The calculated activation for C5 moves from 128 to 136 ppm and the latter value is outside the area of peak increase, so it is possible to state that it does not occur or that it occurs only in a very limited way. The calculated C8 position shifts from 97 to 111 (or 115 for methylene ether bridging) and it is perfectly acceptable; in particular, the amount at 98 ppm of the tannin alone disappears completely in the spectrum of the polymer [35,36]. This means that the position C8 produces results that are almost completely activated. The other positions C6, C2’and C6’ shift from 111–114 to 124–126 and therefore, the three can be involved in methylene or methylene-ether bridges or the simple –CH_2_–OH activation. Even if the pyrogallic ring is far less reactive than the resorcinolic one, the excess of formaldehyde and the long heat exposure allow this electrophilic aromatic substitution as well.

**Figure 4:**
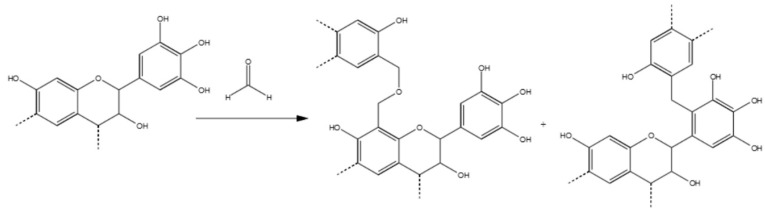
Polymerization reaction of Mimosa tannin with formaldehyde. Methylene-ether and methylene bridges.

In summary, the formaldehyde-polymerized tannins produced methylene-ether and methylene bridges predominantly in the C8, but also in C6, C2’and C6’. The position C5 was not activated. Small portions of unreacted methylol groups, and derived aldehyde and acid, were also observed. Example of the co-polymerization between tannin and formaldehyde are reported in Figure 4.

#### 3.3.2. Tannin–Hexamine (pH = 9.0)

**Figure 5:**
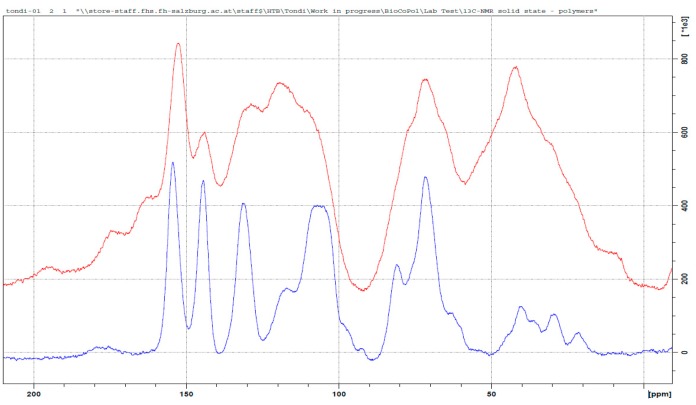
Solid state ^13^C NMR of the original tannin extract (blue) and the leached tannin–hexamine polymer (red).

Tannin–hexamine polymers have been known for decades as wood adhesives [15,37]. Several applicative studies were developed [38,39,40] and only a few more detailed studies were done to understand the way in which hexamine reacts with tannins [41,42]. These studies were conducted in different polymerization conditions. Pizzi and Tekely presented a model where methylene and benzylamine bridges were established, and Pichelin et al. explained that the crosslinking occurs through the formation of reactive imines, and the study of Pena et al. confirmed these findings. In Figure 5 the spectrum of the tannin–hexamine polymer is compared with that of the industrial tannin extract.

The two major absorptions in the area between 90 to 60 and 60 and 20 ppm are related to the non-aromatic part of the polymer. In particular, these two regions can be attributed to hexamine derived bridges: simpler di-benzylamine bridges in which linear crosslinks are involved, such as φ–CH_2_–NH–CH_2_–φ and also longer ones, such as φ–CH_2_–NH–CH_2_–NH–CH_2_–φ, and even slightly branched (but not cyclic) crosslinks are responsible for the broad signal at high fields (20–60 ppm); when the hexamine opens partially and remain cyclic, these carbon atoms absorb at lower field between 90 and 60 ppm. The differently opened hexamine adducts present NH– groups which can also be directly connected to the phenolic ring (e.g., φ–NH–CH_2_–). Therefore, it is very hard to determine a preferential polymerization pattern, due to the contained absorption in the region of 140 ppm, and so the φ–CH_2_–NH– attached are probably preferred to the φ–NH–CH_2_–. It has to be noted further, that according to Pichelin, –NH–CH_2_–O– groups cannot be excluded, and they also would absorb at around 70 ppm.

Similar to formaldehyde, the very broad signal at around 120 ppm of the OH– free aromatic carbons is shifted to a lower field, because these positions will be substituted. In this case, the hexamine also reacts well in the C8, but there are no positions that can be excluded for electrophilic attack.

The bands at 175 and 165 ppm could be due to ionic arrangements. The signal at 175 ppm is due to the deprotonation of the C7 to C–O– and the signal at 165 is due to the deprotonation of the hydroxyl groups on the B-ring (C3’, C4’ and C5’), and in this broad band, the –N–CH=N– bridges also have to be included. The strong signal decrease at 145 ppm, which typically belongs to the C3’, C4’and C5’, validates their ionic structure, confirming the model proposed by Pichelin et al. [29] and it suggests that this arrangement occurs principally in the pyrogallic ring.

**Figure 6:**
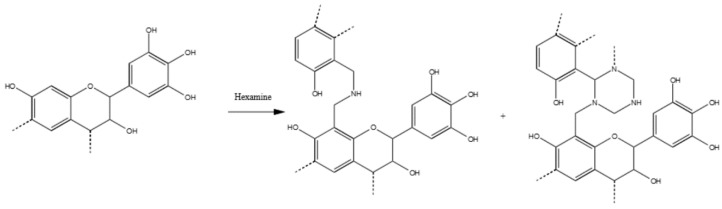
Possible reaction products of tannin–hexamine polymers. Dimethylene amine and cyclic hexamine-derived moieties.

In summary, the polymerization mechanism of tannin and hexamine is very complex and constituted of two parts: The covalent crosslinking which is allowed by various hexamine-derived moieties is principally activated in the ring from the methylene side (less from the amino-side), and to a lesser extent, also in ionic arrangements involving principally the B-ring. In Figure 6, one example of tannin–hexamine polymerization is represented.

#### 3.3.3. Tannin–Glyoxal (pH = 4.5)

**Figure 7:**
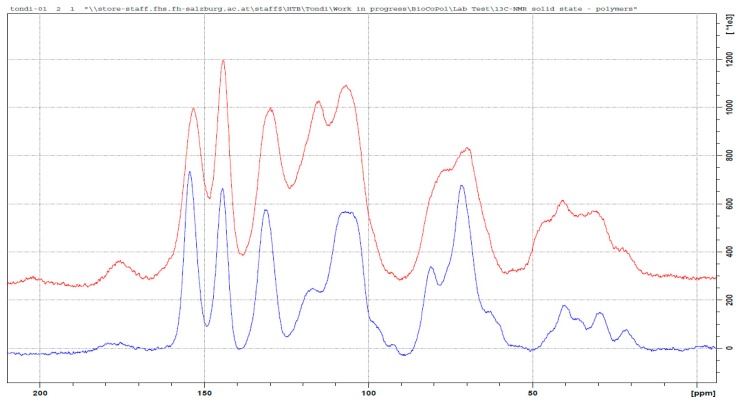
Solid state ^13^C NMR of the original tannin extract (blue) and the leached tannin–glyoxal polymer (red).

The tannin polymers hardened with glyoxal were more recently developed [21], but unfortunately, no detailed studies have been done in order to understand the polymerization mechanism. In Figure 7, the spectra of the tannin–glyoxal polymer is compared with that of the original tannin extract.

Apparently, the reaction seems easy: the dialdehyde reacts with the two functional groups in the activated positions of the flavonoid, producing a diolic crosslink (–CH(OH)–CH(OH)–) or its subsequent enol (–CH=C(OH)– ↔ –CH_2_(–C=O)–), as proposed by Ramirez et al. for phenolic adducts [43]. These two arrangements explain the majority of the peak in the spectrum of Figure 7. Principal conformation of the glyoxal-polymerized tannin is the φ–C(OH)=CH–φ, with very limited occurrence of the keto–enolic equilibrium, because a very small C=O bond is observed (at 205 ppm). The diole structure also occurs and it explains the increase of the signal at around 75 ppm. The increase of the signal at 145 ppm is due to glyoxal only when one of the functional group reacts. Hence, the presence of partially reacted glyoxal is not negligible. In the region 50 to 0 ppm, the patterns are similar to the ones of the tannin powder. Only a new signal at 45 ppm can be observed and it could be due to the formation of a contained number of glyoxal chains e.g., O=C–CH_2_–CHOH–CH–.

In this case the shift of the C_arom_ region is contained, and only a considerable increase of the signal at around 115 ppm can be observed. This suggests that the electrophilic substitution occurs, to a more limited extent, in the same way as it does for formaldehyde and hexamine, and that the C8 position is exclusively preferred.

**Figure 8:**
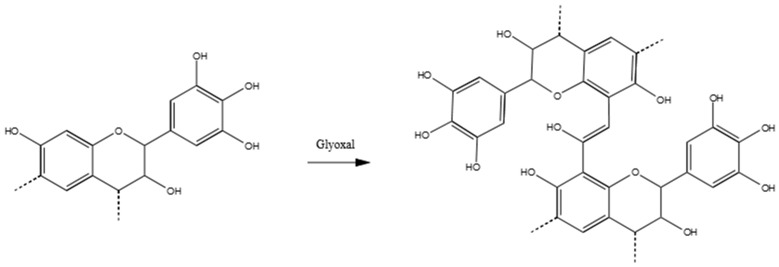
Possible reaction product of tannin–glyoxal polymers.

The majority of the crosslinks in the tannin–glyoxal polymer can be represented by the following schema in Figure 8.

#### 3.3.4. Tannin–Maleic Anhydride (pH = 4.5)

**Figure 9:**
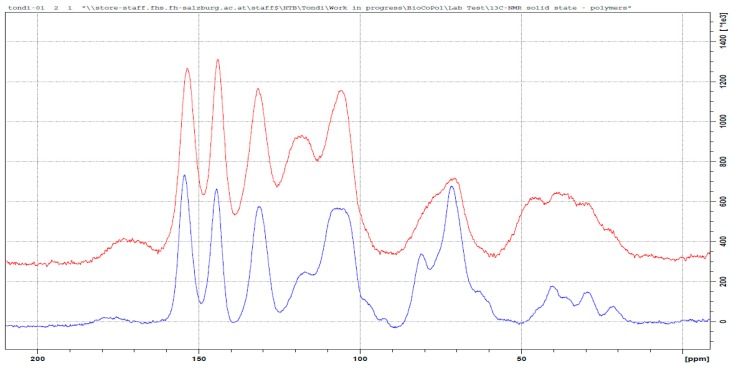
Solid state ^13^C NMR of the original tannin extract (blue) and the leached tannin–maleic anhydride polymer (red).

The polymer between tannin and maleic anhydride was not described before in the literature and its ^13^C NMR spectra is presented in Figure 9. Esterification reactions between tannin and organic acids, such as acetic and propionic acid in 2-pentanone, were proposed to increase the hydrophobicity of the tannin [23]. In our case, the reaction occurs in water, but still, some interesting similarities can be observed.

**Figure 10:**
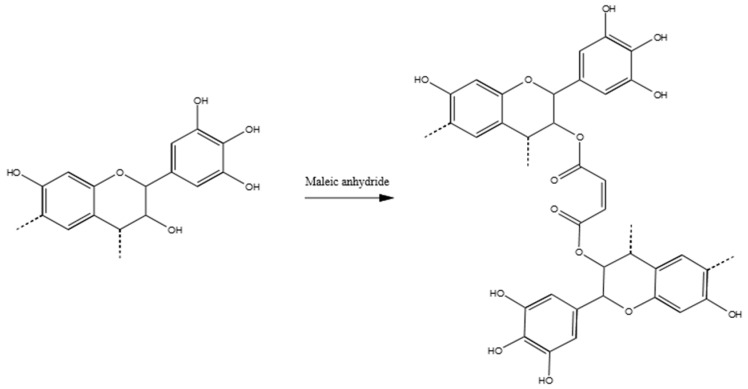
Possible reaction product of tannin–maleic anhydride polymers.

For instance, the new signal at around 175 ppm has to be attributed to the carboxyl group of the ester. In contrast to the other hardeners, this time the increased signal at 120 ppm is due to the C=C double bond of the maleic anhydride, and no shift to a low field is observed (no aromatic ring activation). The broad signal at 75 ppm is due to the C3, which hydroxyl group results esterified. Therefore, this is the most frequently occurring mechanism. The new set of signals between 40 and 50 ppm could be attributed to the carbon of the Diels–Alder coupling of the resorcinolic ring with the double bond of the maleic anhydride [44], even if it occurs in a contained extent. The major polymerized adduct is represented by Figure 10.

#### 3.3.5. Tannin–Furfural (pH = 9.0)

Furfural–tannin polymers were discovered and studied in the 1980s [24,45] because they represented a way to produce a completely natural polymer. These studies introduced the polymerization of tannin–furfural exploiting the self-polymerization of the aldehyde.

**Figure 11:**
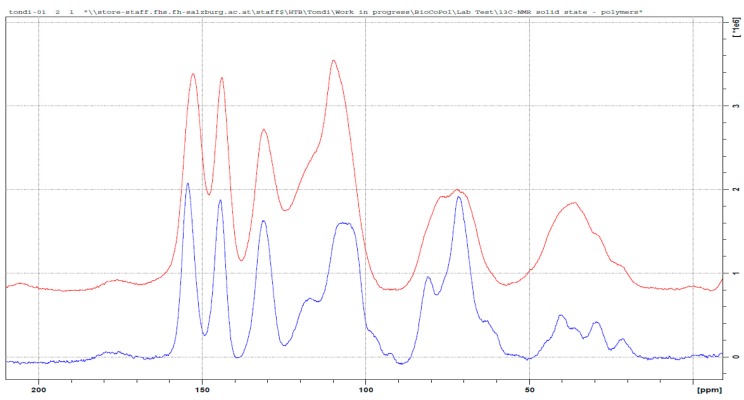
Solid state ^13^C NMR of the original tannin extract (blue) and the leached tannin–furfural polymer (red).

The mechanism proposed by Foo and Hemingway explains all the signals of the spectrum of the tannin–furfural polymer. In Figure 11, the major peak at around 110 ppm is very intense because the C2 and C3 of the furanic ring, while the other two signals are absorbed at 141 and 156 ppm, which overlaps with the aromatic C–O of the tannin. The carbon has the carbonyl result reduced and directly bonds with two resorcinolic rings and is absorbed at ~40 ppm. When the furfural reacts with only one ring, the resulting alcohol absorbtion is at around 70 ppm, which explains the increase of that signal in the spectrum. The furfural can also self-react, producing furanic chains where the methylene group between two furanic rings absorbs at around 30 ppm, and the side with unreacted carbonyl absorbs at 175 ppm. These chains can also develop in the Diels–Alder arrangement, which would confirm the signal increase at high fields. Also, for this co-monomer, a shift in the signal of the aromatic C–H is observed, which suggests the activations of these positions. The amount over 200 ppm can be due to a few of the furanic rings that open producing carbonyl groups. The related methylene groups due to this opening contributes to the signal at around 40 ppm [46].

**Figure 12:**
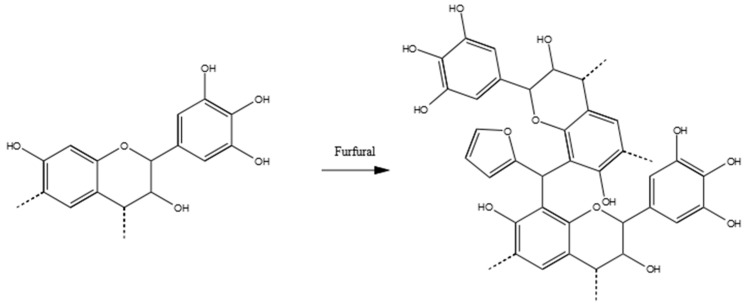
Possible reaction product of tannin–furfural polymers.

Figure 12 summarises the more probable polymer of tannin–furfural results, where the reduced methylene group of furfural produces a bridge between tannin oligomers. However, chains of furfural which can present the tridimensional Diels–Alder arrangement and furanic ring opening, cannot be excluded.

#### 3.3.6. Tannin–Furfuryl Alcohol (pH = 2.0)

**Figure 13:**
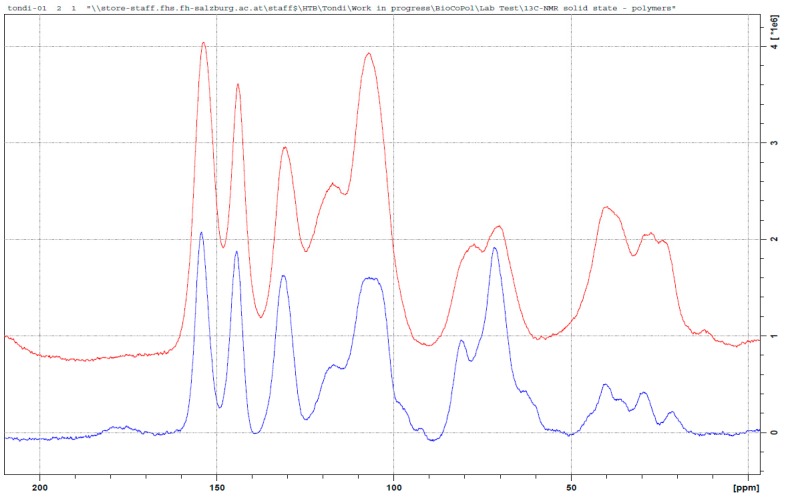
Solid state ^13^C NMR of the original tannin extract (blue) and the leached tannin–furfuryl alcohol polymer (red).

In Figure 13, the tannin–furfuryl alcohol spectrum appears to be very similar to that of tannin–furfural. These polymers are not new in the literature because they are constituted of adducts which were already considered for rigid foams and adhesives [47,48,49,50]. In these studies, the intimate interconnection between tannin and furanic moieties was established and the presence of the Diels–Alder rearrangement was also proposed, but until now, no ^13^C NMR study has been done [51,52].

In this case, the presence of the furanic ring can be easily observed by the signal in regions around 110, 145 and 155 ppm. The major difference between this spectrum and that of the tannin–furfural previously discussed is the presence of a more intense band at high field 30–10 ppm which can be attributed to a more favoured Diels–Alder rearrangement by the acidic environment. The presence of some –OH in the polyfurfuryl chain would be absorbed at around 75 ppm, which is also not new for these materials [53].

The presence of the high signal at 110 ppm hides the increase of the phenolic activation; however, if we compare the relative intensities of the peaks at 120 with that at 130 ppm, it appears clearly that in this case the electrophilic aromatic also substitution occurs. In this case too, the furanic ring opening cannot be excluded, due to the presence of the signal at a very low field (210 ppm).

**Figure 14:**
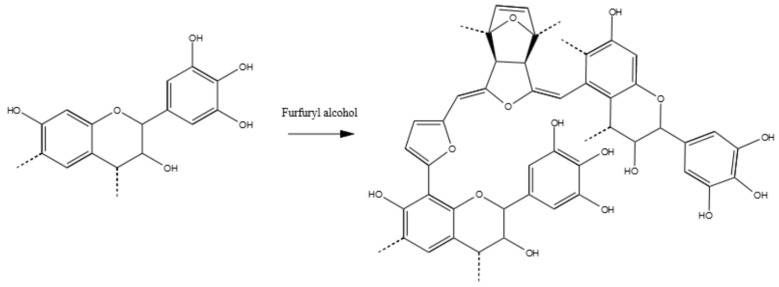
Possible reaction product of tannin–furfuryl alcohol polymers.

In summary, the activation through furfuryl alcohol seems to be similar to that of furfural, with a more significant amount of Diels–Alder arrangements and/or ring openings. Figure 14 shows a possible reaction product.

It is worth noting that for every polymer there is a general reduction of the peak at around 72 ppm and the disappearance of the small shoulder at 92 ppm, which can be attributed to the loss of easy sugars after leaching.

### 3.4. FT–IR Spectroscopy

**Figure 15:**
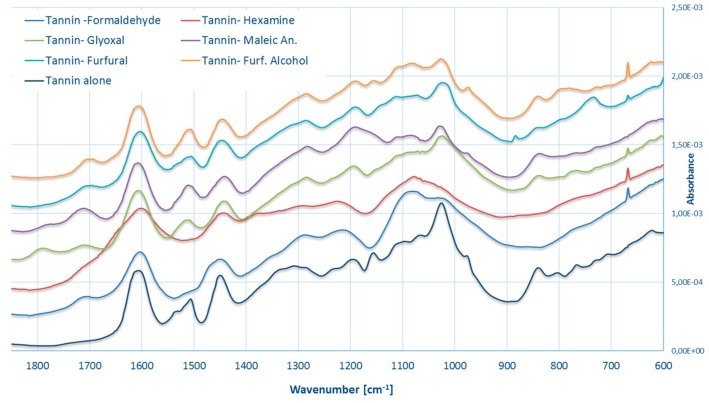
FT-IR spectra of the different leached tannin-based solids.

The spectra of the industrial tannin powder and that of the six possible polymers are reported in Figure 15. The infrared region between 1800 and 600 cm^−1^ is the most significant for tannin extracts and it can be interpreted by separating it in four different regions [54]:

C=O region (1800–1650 cm^−1^): In this region there are the absorbances of the C=O stretching. All of the powders present some absorbance in this region and the “activated” tannins show a significant increase, which must be due to nature or to the interaction of the hardener with tannin. For the reaction model proposed for maleic anhydride and glyoxal, the C=O group is due to the moiety itself; for furfural and furfuryl alcohol it is due to the furan ring opening, while for hexamine this is due to the broad stretching of the secondary amines [55]. In the tannin–formaldehyde copolymer, this signal should be due to the presence of oxided methylol branches.

Aromatic region (1650–1400 cm^−1^): The region at around 1600 and 1500 cm^−1^ is typically due to aromatic C=C stretching and it looks relatively similar for every powder except tannin–formaldehyde and tannin–hexamine. These two show limited absorptions at around 1500 cm^−1^ which could be due to possible delocalization of the π electrons, when the structure becomes polymerized. Another possibility is due to the shifting of the signal to around 1450 cm^−1^ which, indeed, appears broader for every tannin–hardener formulation and it can be assigned to several transitions involving aromatic atoms: C-H bending as well as C–O and C–C stretching are all possible. The broader profiles of this spectral area suggest some similarity between the “activated formulations”.

C_arom_–O region (1400–1100 cm^−1^): The band at around 1350 cm^−1^ that can be attributed to the C–O stretching of pyrogallic moieties become less intense when hardeners are added, except for the formaldehyde cured resin. The signal at 1160 cm^−1^ decreases/disappears for formaldehyde, hexamine, glyoxal and furfural, while it resists in the case of maleic anhydride and furfuryl alcohol. This signal has to be attributed to C–O stretching and C–C bending of aromatics. Furthermore, formaldehyde and hexamine present a broader peak in the region close to 1100 cm^−1^, due to C–H bending of aromatic. These observations might be attributed to sterical hindrance of the polymerized molecule. In particular, the new tannin structures obtained with hexamine and formaldehyde may result as tightly interconnected.

Low wave number region (1100–600 cm^−1^): Some information can be observed also in this region. The signal close to 1040 cm^−1^ is lower and broader for every hardener; the signal at 980 cm^−1^ disappears for every formulation, except for maleic anhydride and furfuryl alcohol. The signal at 840 cm^−1^ decreases for every formulation and disappears for formaldehyde and hexamine. Finally, a small new signal at 680 cm^−1^ can be observed for all formulations except maleic anhydride. These signals are very difficult to interpret, but they can also participate in defining different families of adduct.

According to this line of thought, we can clearly distinguish the “highly cross-linked” polymers constituted by the tannin–formaldehyde and the tannin–hexamine formulations, which present no/low signal at 1500 cm^−1^, the broader profile in the region around 1450 cm^−1^ as well as a smooth profile at higher frequencies, which is due to the compacted arrangement of the tannin moieties. The other hardeners can be all classified as “low cross-linked” even if some small differences can be highlighted.

**Figure 16:**
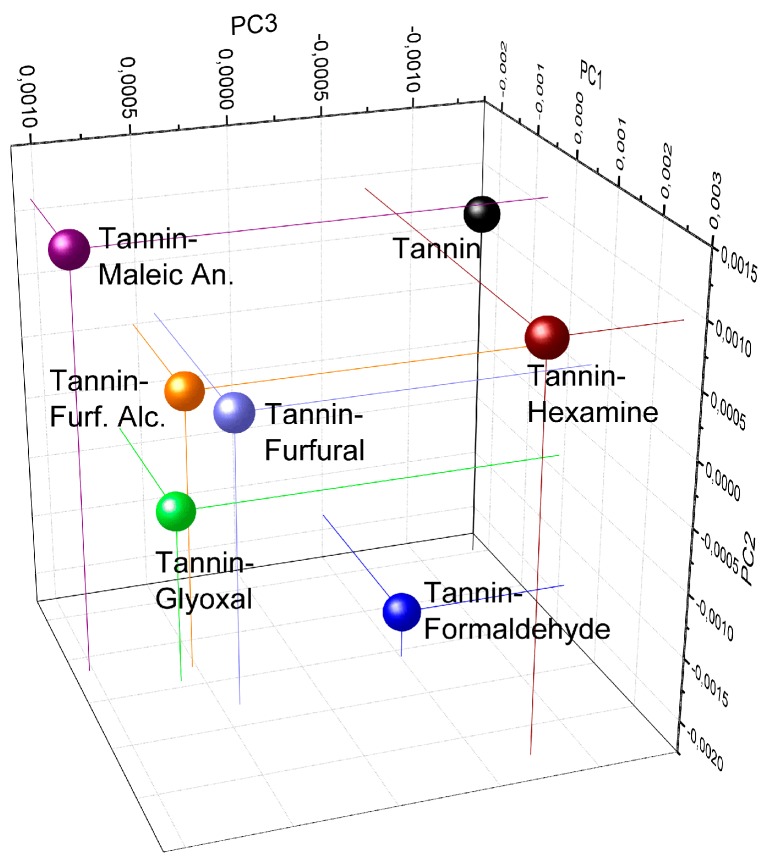
Principal component analysis of the FT-IR spectra of the industrial tannin extract in comparison with the six tannin-based copolymers.

The principal component analysis can be exploited to unscramble the classification. In Figure 16 the three principal components for every powder are presented in a 3D graphic.

We can observe that the “high-crosslinked” copolymers and the tannin extract have negative PC3 while the “low cross-linked” stay relatively close in the same PC3-positive area. Even if the spectra of the first group present a similar value to that of PC3, they are very different because they are located in three different regions: the tannin extract presents negative PC1 and positive PC2; formaldehyde shows negative PC2; and hexamine has positive PC1 and PC2. Conversely, the “low cross-linked” polymers have similar PC1 and PC3 and they appear more similar to tannin–formaldehyde at least in terms of PC1 values. A suggestive interpretation of these results can be the following.

High values of PC1 and/or PC2 might be related to high molecular masses/high cross-linking degree. This observation can be done also on the basis of the strong modification of the region between 140 and 90 ppm of the ^13^C NMR spectra, and on the very broad profile of the FT-IR spectra of the tannin–formaldehyde and tannin–hexamine copolymers. High values of PC2 and/or PC3 might be related to the length of the branches at the consequent degree freedom of the copolymer between chains. This explanation is suggested by observing that formaldehyde produces short methylene or methylene-ether bridges and registers relative low values for PC2 and PC3, while hexamine and maleic anhydride produces complex –NH–CH_2_– chains and 6-atomic (ring opening) bridges as well as high PC2 scores.

## 4. Conclusions

Several Mimosa tannin copolymers have been produced by simple hardening a 33% tannin solution with different monomers, at various concentration, pH and temperature conditions. The formulation that resulted as solid after the heating phase was than leached to establish which of the outcomes was really polymerized. Six hardeners, namely formaldehyde, hexamine, glyoxal, maleic anhydride, furfural and furfuryl alcohol produced effective results and the tannin–hardener polymers were then analysed by solid state ^13^C NMR and FT-IR to identify the product of the reactions.

Formaldehyde: The polymer that resulted was highly water resistant and the polymerization through methylene and methylene–ether bridges was confirmed. The prorobinetinidin was highly activated and the only aromatic position that was unaffected was the C5, while the C8 was completely activated. A small portion of unreacted methylol groups was also noticed. The FT-IR suggests that the copolymer is highly crosslinked.

Hexamine: The copolymer of tannin–hexamine is very hard to understand because complicated activations were observed. The majority of the crosslinking were –CH–NH– chains where sometimes the original 6-term ring remains. However, the ionic arrangement cannot be excluded. Also this copolymer was highly reticulated.

Glyoxal: The spectra of the tannin–glyoxal copolymers can be understood considering two similar cross-linkages: the diolic (–CH(OH)–CH(OH)–) and the enolic (–C(OH)=CH–). These polymers were not very cross-linked and the activation occurred to a lesser extent and with high probability principally in the C8 of the proanthocyanidin. Part of the glyoxal that activated the aromatic ring does not crosslink at all, leaving the second carbonyl group unreacted.

Maleic anhydride: Esterification reaction occurred between the tannin and maleic anhydride. This esterification may occur in different hydroxyl groups, but the more probable is the aliphatic –OH in position 3. Some evidence of the Diels–Alder arrangement were also observed in the ^13^C-NMR. The FT-IR spectrum of this copolymer also shows low cross-linking patterns.

Furfural: The polymerization of furfural is well-explained by previous studies, where the carbonyl group results directly combined with two phenolics. However, the furfural can also produce chains where the heterocycle can further explain the presence of the carbonyl group. Additionally, this copolymer was low cross-linked.

Furfuryl alcohol: In this polymer the polymerization involved a significant amount of homo-polymerization of furfuryl alcohol that combined with the tannin oligomers into complex structures. Additionally, in this case, the opening of the furanic ring occurred and Diels–Alder arrangement occurred. The FT-IR suggests that this polymer has a limited crosslinking degree.

## Figures and Tables

**Table 1:** Design of curing experiment: Hardener, amount and pH.

Family of Hardeners	Hardener	Amount (% s./s. tannin)	pH
Known	Formaldehyde, Hexamine	4%, 6%, 8% and 25%	2, 4.5, 7, 9
Aldehydes	Glyoxal, Glutaraldehyde, *p*-phthaldialdehyde	6%, 12.5%, 25% and 50%	2, 4.5, 7, 9
Nitrogen compound	Caprolactam, Dicyandiamide, Chitosan	6%, 12.5%, 25% and 50%	2, 4.5, 7, 9
Acids	Citric acid, Maleic anhydride, Phthalic acid	6%, 12.5%, 25% and 50%	2, 4.5, 7, 9
Furanics	Furfural, Furfuryl alcohol	6%, 12.5%, 25% and 50%	2, 4.5, 7, 9

**Table 2:** Set of polymerization experiments of tannin solutions with different hardeners, amounts, pH levels and temperatures.

Hardener	Amount	pH	Hardening Temp. (°C)	Observations
Formaldehyde	4%, 6%, 8%; 25%	Every	20 to 103	pH 2 and 9 hardens at lower *T*
Hexamine	4%, 6%, 8%; 25%	9, 7, 9	50 to 90	Homogeneous polymers
Glyoxal	12.5%, 25%	Every	90 to 103	Elastic solids
Glutaraldehyde	50%	9	70	Weak gel
*p*-Phthaldialdehyde	12.5%	9	50	Two phases
Caprolactam	Any	Any	No hardening	Remains liquid
Chitosan	Any	Any	No hardening	Remains liquid/clumps
Dicyandiamide	Any	Any	No hardening	Remains liquid
Citric acid	Any	Every	No hardening	Remains liquid
Maleic anhydride	12.5% to 50%	2, 4.5	100	Elastic solid
Phthalic acid	Any	Every	No hardening	Remains liquid
Furfural	25%, 37.5%	Every	103, 70, 50	High amount/pH → Solid
Furfuryl alcohol	12.5%, 25%	2, (4.5)	90, 103	Hard, black solid

## References

[1] Douglas G.B., Wang Y., Waghorn G.C., Barry T.N., Purchas R.W., Foote A.G., Wilson G.F. (1995). Liveweight gain and wool production of sheep grazing Lotus corniculatus and lucerne (Medicago sativa). N. Z. J. Agric. Res..

[2] Meier H., Buchs L., Buchala A.J., Homewood T. (1981). (1→3)-β-d-Glucan (callose) is a probable intermediate in biosynthesis of cellulose of cotton fibres. Nature.

[3] Woodings C. (2001). Regenerated Cellulose Fibres.

[4] Arbenz A., Avérous L. (2015). Chemical modification of tannins to elaborate aromatic biobased macromolecular architectures. Green Chem..

[5] Pizzi A. (1979). Phenolic and tannin-based adhesive resins by reactions of coordinated metal ligands. II. Tannin adhesive preparation, characteristics, and application. J. Appl. Polym. Sci..

[6] Bisanda E.T.N., Ogola W.O., Tesha J.V. (2003). Characterisation of tannin resin blends for particle board applications. Cem. Concr. Compos..

[7] Pizzi A. (2006). Recent developments in eco-efficient bio-based adhesives for wood bonding: Opportunities and issues. J. Adhes. Sci. Technol..

[8] Jahanshahi S., Pizzi A., Abdulkhani A., Shakeri A. (2016). Analysis and testing of bisphenol A—Free bio-based tannin epoxy-acrylic adhesives. Polymers.

[9] Khanbabaee K., van Ree T. (2001). Tannins: classification and definition. Nat. Prod. Rep..

[10] Bate-Smith E.C., Swain T. (1962). Flavonoid compounds. Comp. Biochem..

[11] Beckman C.H. (2000). Phenolic-storing cells: Keys to programmed cell death and periderm formation in wilt disease resistance and in general defence responses in plants?. Physiol. Mol. Plant Pathol..

[12] Sen S., Tascioglu C., Tırak K. (2009). Fixation, leachability, and decay resistance of wood treated with some commercial extracts and wood preservative salts. Int. Biodeterior. Biodegrad..

[13] Zhan K., Ejima H., Yoshie N. (2016). Antioxidant and adsorption properties of bioinspired phenolic polymers: A comparative study of catechol and gallol. ACS Sustain. Chem. Eng..

[14] Haslam E. (1989). Plant Polyphenols: Vegetable Tannins Revisited.

[15] Pizzi A. (1994). Advanced Wood Adhesives Technology.

[16] Pasch H., Pizzi A., Rode K. (2001). MALDI–TOF mass spectrometry of polyflavonoid tannins. Polymer.

[17] Hemingway R.W., Karchesy J.J. (2012). Chemistry and Significance of Condensed Tannins.

[18] Pizzi A., Mittal K.L. (2011). Wood Adhesives.

[19] Theis M., Grohe B. (2002). Biodegradable lightweight construction boards based on tannin/hexamine bonded hemp shaves. Holz als Roh und Werkstoff.

[20] Kain G., Güttler V., Barbu M.C., Petutschnigg A., Richter K., Tondi G. (2014). Density related properties of bark insulation boards bonded with tannin hexamine resin. Eur. J. Wood Wood Prod..

[21] Ballerini A., Despres A., Pizzi A. (2005). Non-toxic, zero emission tannin-glyoxal adhesives for wood panels. Holz als Roh und Werkstoff.

[22] Santiago-Medina F.J., Pizzi A., Basso M.C., Delmotte L., Celzard A. (2017). Polycondensation Resins by Flavonoid Tannins Reaction with Amines. Polymers.

[23] Grigsby W.J., Bridson J.H., Lomas C., Elliot J.A. (2013). Esterification of condensed tannins and their impact on the properties of poly (lactic acid). Polymers.

[24] Foo L.Y., Hemingway R.W. (1985). Condensed tannins: Reactions of model compounds with furfuryl alcohol and furfuraldehyde. J. Wood Chem. Technol..

[25] Hauptmann M., Gindl-Altmutter W., Hansmann C., Bacher M., Rosenau T., Liebner F., Schwanninger M. (2015). Wood modification with tricine. Holzforschung.

[26] Banfi D., Patiny L. (2008). Resurrecting and Processing NMR Spectra On-line. CHIMIA Int. J. Chem..

[27] Castillo A.M., Patiny L., Wist J. (2011). Fast and accurate algorithm for the simulation of NMR spectra of large spin systems. J. Magn. Reson..

[28] Steinbeck C., Krause S., Kuhn S. (2003). NMRShiftDB constructing a free chemical information system with open-source components. J. Chem. Inf. Comput. Sci..

[29] Pichelin F., Kamoun C., Pizzi A. (1999). Hexamine hardener behaviour: Effects on wood glueing, tannin and other wood adhesives. Eur. J. Wood Wood Prod..

[30] Pilato L. (2010). Phenolic Resins: A Century of Progress.

[31] Roux D.G., Ferreira D., Hundt H.K., Malan E. (1975). Structure, stereochemistry, and reactivity of natural condensed tannins as basis for their extended industrial application. Appl. Polym. Symp..

[32] Grenier-Loustalot M.F., Larroque S., Grenier P., Bedel D. (1996). Phenolic resins: 4. Self-condensation of methylolphenols in formaldehyde-free media. Polymer.

[33] Rego R., Adriaensens P.J., Carleer R.A., Gelan J.M. (2004). Fully quantitative carbon-13 NMR characterization of resol phenol–formaldehyde prepolymer resins. Polymer.

[34] Pizzi A., Stephanou A. (1993). A comparative C13 NMR study of polyflavonoid tannin extracts for phenolic polycondensates. J. Appl. Polym. Sci..

[35] Pizzi A., Scharfetter H.O. (1978). The chemistry and development of tannin-based adhesives for exterior plywood. J. Appl. Polym. Sci..

[36] Kiatgrajai P., Wellons J.D., Gollob L., White J.D. (1982). Kinetics of polymerization of (+)-catechin with formaldehyde. J. Org. Chem..

[37] Pizzi A., Valenezuela J., Westermeyer C. (1994). Low formaldehyde emission, fast pressing, pine and pecan tannin adhesives for exterior particleboard. Eur. J. Wood Wood Prod..

[38] Thevenon M.F., Tondi G., Pizzi A. (2009). High performance tannin resin-boron wood preservatives for outdoor end-uses. Eur. J. Wood Wood Prod..

[39] Szczurek A., Fierro V., Pizzi A., Stauber M., Celzard A. (2014). A new method for preparing tannin-based foams. Ind. Crop. Prod..

[40] Pena C., De la Caba K., Retegi A., Ocando C., Labidi J., Echeverria J., Mondragon I. (2009). Mimosa and chestnut tannin extracts reacted with hexamine in solution. J. Therm. Anal. Calorim..

[41] Pichelin F., Nakatani M., Pizzi A., Wieland S. (2006). Structural beams from thick wood panels bonded industrially with formaldehyde-free tannin adhesives. For. Prod. J..

[42] Pizzi A., Tekely P. (1995). Mechanism of polyphenolic tannin resin hardening by hexamethylenetetramine: CP–MAS 13C NMR. J. Appl. Polym. Sci..

[43] Ramires E.C., Megiatto J.D., Gardrat C., Castellan A., Frollini E. (2010). Biobased composites from glyoxal–phenolic resins and sisal fibers. Bioresour. Technol..

[44] Trivedi B. (2013). Maleic Anhydride.

[45] Rossouw D.D.T., Pizzi A., McGillivray G. (1980). The kinetics of condensation of phenolic polyflavonoid tannins with aldehydes. J. Polym. Sci. Polym. Chem. Ed..

[46] Gandini A., Belgacem M.N. (1997). Furans in polymer chemistry. Prog. Polym. Sci..

[47] Abdullah U.H.B., Pizzi A. (2013). Tannin-furfuryl alcohol wood panel adhesives without formaldehyde. Eur. J. Wood Wood Prod..

[48] Luckeneder P., Gavino J., Kuchernig R., Petutschnigg A., Tondi G. (2016). Sustainable Phenolic Fractions as Basis for Furfuryl Alcohol-Based Co-Polymers and Their Use as Wood Adhesives. Polymers.

[49] Link M., Kolbitsch C., Tondi G., Ebner M., Wieland S., Petutschnigg A. (2011). Formaldehyde-free tannin based foams and their use as lightweight panels. BioResources.

[50] Basso M.C., Pizzi A., Lacoste C., Delmotte L., Al-Marzouki F.M., Abdalla S., Celzard A. (2014). MALDI-TOF and 13C NMR analysis of tannin-furanic-polyurethane foams adapted for industrial continuous lines application. Polymers.

[51] Tondi G., Link M., Oo C.W., Petutschnigg A. (2015). A simple approach to distinguish classic and formaldehyde-free tannin based rigid foams by ATR FT-IR. J. Spectrosc..

[52] Reyer A., Tondi G., Berger R.J.F., Petutschnigg A., Musso M. (2016). Raman spectroscopic investigation of tannin-furanic rigid foams. Vib. Spectrosc..

[53] Pizzi A., Tondi G., Pasch H., Celzard A. (2008). Matrix-assisted laser desorption/ionization time-of-flight structure determination of complex thermoset networks: Polyflavonoid tannin–furanic rigid foams. J. Appl. Polym. Sci..

[54] Tondi G., Petutschnigg A. (2015). Middle infrared (ATR FT-MIR) characterization of industrial tannin extracts. Ind. Crop. Prod..

[55] Coates J. (2000). Interpretation of infrared spectra, a practical approach. Encyclopedia of Analytical Chemistry.

